# A case report of *Emergomyces orientalis* infection in the lung of HIV infected in patient and literature review

**DOI:** 10.3389/fmed.2026.1874569

**Published:** 2026-08-26

**Authors:** Lifeng Luo, Ying Guo, Zunzhen Nie

**Affiliations:** 1 Department of Thoracic Surgery, Xi’an Daxing Hospital Affiliated to Yan’an University, Xi’an, China; 2 Department of Pathology, Xi’an Daxing Hospital Affiliated to Yan’an University, Xi’an, China

**Keywords:** antifungal treatment, *Emergomyces orientalis*, HIV infected, metagenomic next-generation sequencing, pulmonary infection

## Abstract

**Objective:**

This study reports a rare case of pulmonary *Emergomyces orientalis* infection with clinical characteristics, diagnostic approaches and therapeutic regimens of this rare fungal infection, aiming to provide evidence for clinical diagnosis and management.

**Methods:**

We retrospectively analyzed the patient’s clinical symptoms, imaging manifestations, laboratory examinations including metagenomic next-generation sequencing (mNGS), diagnostic and therapeutic procedures, as well as prognosis. Related published literature was searched, reviewed and summarized.

**Results:**

The patient presented with a 3-day cough and expectoration. Conventional pathogen examinations showed the patient is infected with the HIV virus and definite diagnosis of *Emergomyces orientalis* infection was finally confirmed by mNGS of lung biopsy specimens. He received liposomal amphotericin B combined with symptomatic supportive therapy. Clinical symptoms were alleviated and pulmonary lesions were absorbed after treatment.

**Conclusion:**

Pulmonary *Emergomyces orientalis* infection is uncommon and prone to missed diagnosis and misdiagnosis with high mortality. Routine pathogen culture yields a low positive rate. mNGS serves as a critical tool for early and accurate diagnosis. Individualized antifungal treatment, strict drug interaction assessment and therapeutic monitoring are essential to optimize clinical prognosis.

## 1 Introduction

*Emergomyces orientalis* is a rare thermally dimorphic endemic fungus predominantly prevalent in China, which mainly causes pulmonary and disseminated *emergomyces*. The fungus is reported in a 64-year-old male of Type 2 Diabetes Mellitus from Shanxi, China (1). Most reported infections occur in immunocompromised hosts, while cases involving immunocompetent individuals are extremely scarce and easily misdiagnosed as pulmonary tuberculosis or other fungal infections due to nonspecific clinical and imaging manifestations. While primarily associated with immunocompromised individuals, especially those with HIV (2). Previously, a retrospective study from southern Africa assessed 54 patients with disseminated *emergomyces*, of whom 94% were co-infected with HIV; 96% had skin involvement, 88% had lung involvement, 44% received an incorrect diagnosis, and 48% died (3) early diagnosis remains challenging owing to limited clinical awareness and low detection rate of conventional microbial tests. Herein, we report a rare case of isolated pulmonary *Emergomyces orientalis* infection in an HIV infected patient, summarize clinical characteristics, diagnostic strategies and therapeutic outcomes, and review relevant literature to improve clinicians’ recognition and management of this rare fungal pathogen.

## 2 Case presentation

### 2.1 General circumstances

The patient is a 38-year-old male inpatient. His occupation is clerk and as Han ethnicity. After admission, the laboratory tests showed: CD4+/CD8+ = 0.09; HIV viral load > 300 copies/106 PRMC. The initial diagnosis of HIV infection was made after laboratory tests upon admission. The patient was informed about the use of ART, but refused. The patient has been working in a restaurant for a long time, mainly in the kitchen and the lobby, without engaging in agricultural work, and has no history of exposure to soil or dust. There is no travel history, contact with animals, or living environment. The patient denies long-term use of immunosuppressants or history of organ transplantation. There is no history of smoking or drinking, and also denies a history of drug allergies.

### 2.2 Clinical manifestation

Three days ago, the patient developed a cough with no obvious cause. The cough was paroxysmal and dry, with occasional expectoration of a small amount of white mucus sputum. There was no hemoptysis, chest pain, or chest tightness. The patient also had no fever, chills, or rigors; no fatigue, night sweats, or weight loss; and no nausea, vomiting, or abdominal pain. On admission, a physical examination by a specialist revealed clear bilateral lung sounds without any dry or wet rales. The heart rate was 82 beats per minute and regular, and no pathological murmurs were heard in the auscultation areas of the heart valves.

### 2.3 Auxiliary examination

Sputum smear Gram staining: No bacteria or fungal spores were detected, and acid-fast staining was negative.

The fungal cultures conducted in our hospital was negative (culture medium: Sabouraud Dextrose Agar).

Sputum culture (culture medium: Chocolate Agar): No pathogenic bacteria grew.

**FIGURE 1:**
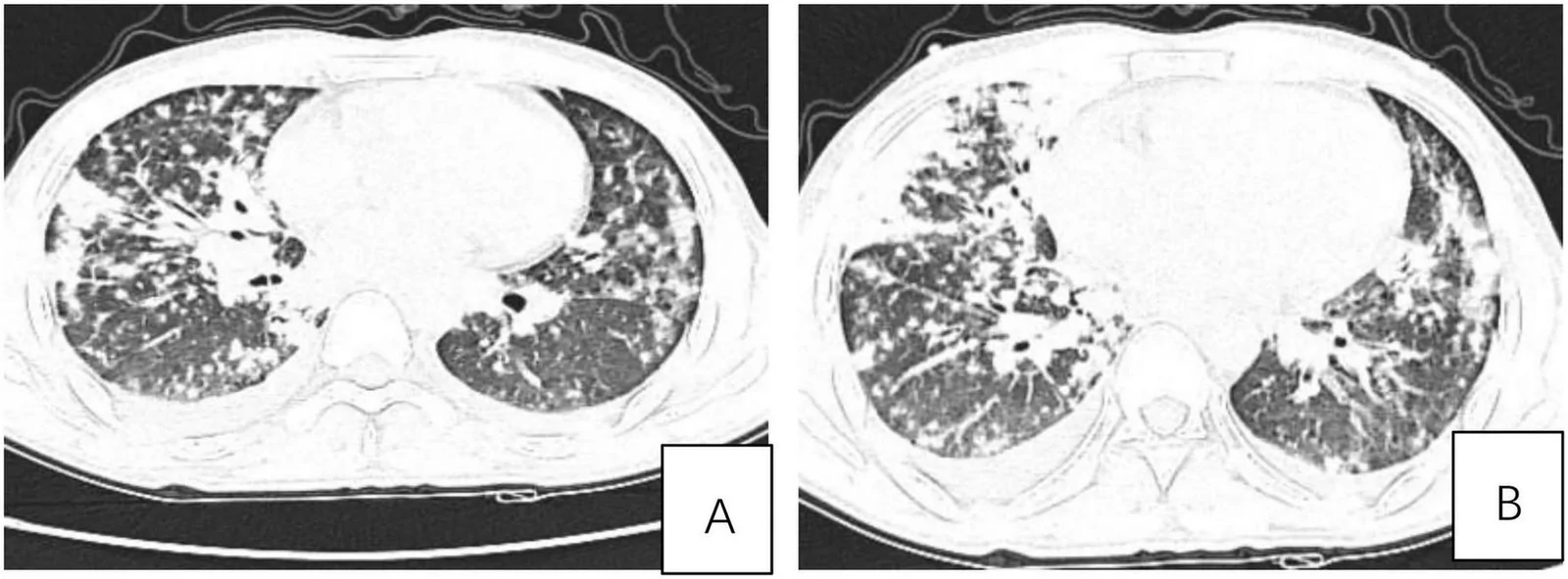
**(A,B)** Plain chest CT scan: Patchy ground-glass opacities and a few areas of consolidation were seen in the lower lobe of the right lung, with ill-defined borders. Inflammatory lesions were suspected.

Plain chest CT scan revealed patchy ground-glass opacities and a few consolidation shadows in the lower lobe of the right lung, with unclear boundaries. Inflammatory lesions were considered (Figure 1).

**FIGURE 2:**
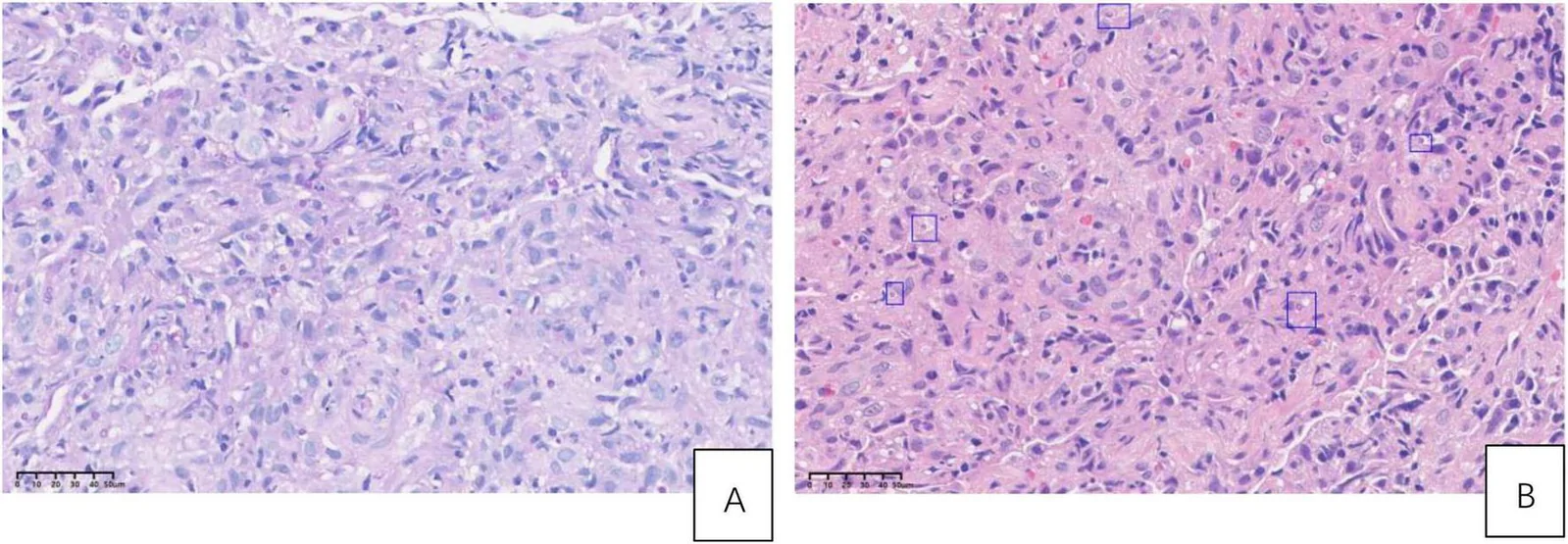
**(A)** Interstitial lung hyperplasia, with lymphocyte infiltration observed in the alveolar interstitium, and cells with inclusion bodies, presenting eosinophilic characteristics, are shown in panel **(A)**. **(B)** PAS staining: a few faint yeast-like fungal spores were detected.

To clarify the etiological diagnosis, a fiberoptic bronchoscopy was performed, and lung tissue biopsy was performed for pathological examination (Figure 2).

mNGS testing (DNA sequencing) through high-throughput sequencing and intelligent algorithm analysis, can detect 21,388 types of microorganisms, including 11,958 types of bacteria (including 180 types of mycobacteria, 251 types of chlamydia/mycoplasma/rickettsia/spirochetes), 7,373 types of viruses (including 4,414 types of RNA viruses, 2,959 types of DNA viruses), 1,714 types of fungi and 343 types of parasites, with a sequencing depth of ≥ 5 million clean reads after host removal and comparison with microbial genome databases revealed the presence of the specific sequence of Emergomyces orientalis, which include 1664 specific sequences, with an abundance of 69.47% and a coverage of 0.2%,and no other pathogenic bacteria or drug resistance genes were detected.

### 2.4 Diagnosis and treatment, follow-up

**FIGURE 3:**
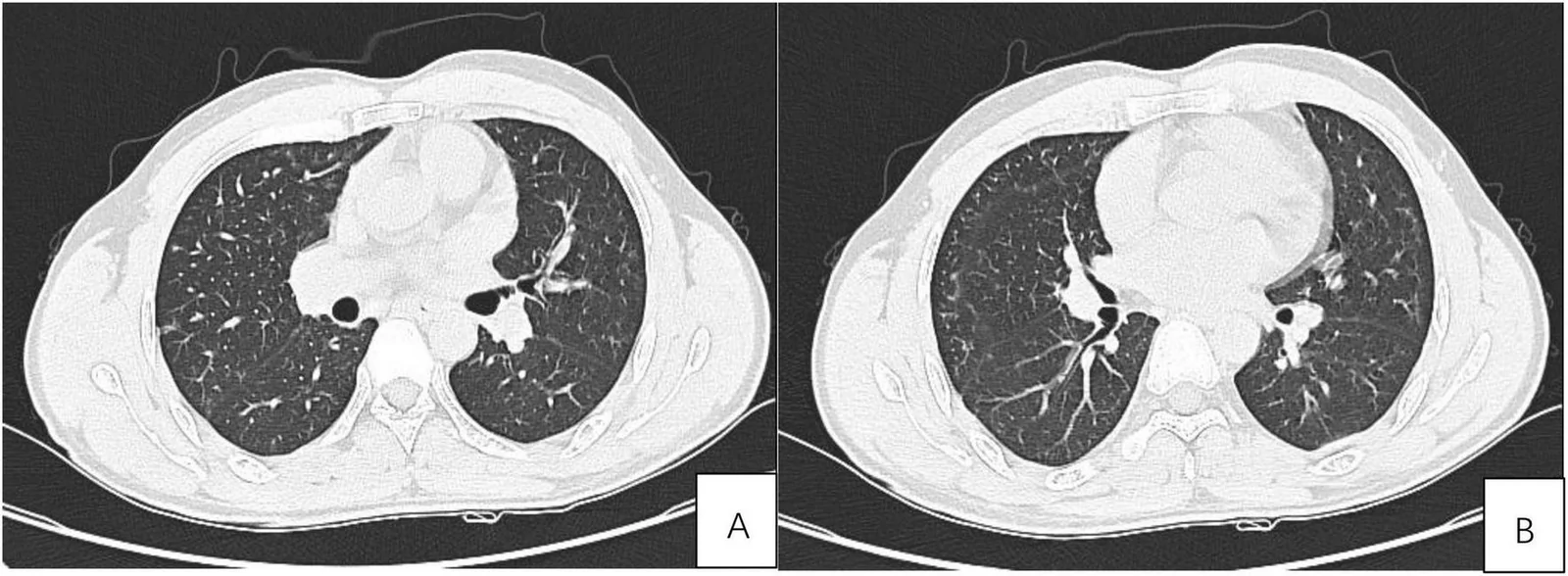
**(A,B)** The transparency of both lungs is basically symmetrical, and no diffuse pulmonary emphysema or large patchy consolidation shadows are observed. Diffuse and randomly distributed millet-like / tiny nodular shadows can be seen in both lung fields, which is consistent with the post-treatment response.

The final diagnosis was pulmonary infection with Emergomyces orientalis. The treatment was carried out using liposomal amphotericin B combined with symptomatic and supportive therapy. The dosage was 5–10 mg/(kg⋅d), and the treatment lasted for 1 month. During the treatment period, routine blood tests were conducted regularly, and liver and kidney functions were rechecked. No obvious adverse reactions were observed. The symptoms were relieved, and there were no symptoms such as coughing, expectoration, or fever. The patient did not undergo repeated microbiological tests. After 1 month of follow-up by phone, the condition was stable, and there was no recurrence of cough symptoms. A repeat chest CT scan showed that compared with the first CT scan, the patchy ground-glass-like density and consolidation in the lower lobe of the right lung had significantly decreased (Figure 3). The patient is regular follow-up.

### 2.5 Antidiastole

This disease needs to be differentiated from three disease.

#### 2.5.1 Pulmonary histoplasmosis

Pulmonary histoplasmosis belongs to the Histoplasma complex, with highly similar morphological features and imaging manifestations. The yeast cells of Histoplasma are smaller with thicker cell walls. Definitive differentiation relies on fungal culture and nucleic acid testing.

#### 2.5.2 Pulmonary blastomycosis

Both conditions may present with pulmonary nodules, cavities and disseminated lesions. Blastomyces shows broad-based budding, while *Emergomyces* has narrow-necked budding. Differentiation is confirmed by etiological examination and culture.

#### 2.5.3 Pulmonary coccidioidomycosis

They differ in endemic regions. Coccidioides is characterized by spherules and endospores, with distinct microscopic morphology.

## 3 Discussion

*Emergomyces orientalis* is a biphasic fungus that was first identified by Chinese scholars in 2017 and is classified under the genus *Emergomyces*. Only a dozen cases have been reported globally, and this is the first case reported in Xi’an, China (1). Zhou (2) is the first detailed description of a cavitary nodule on conventional chest CT in E. orientalis infection, which differs from previously reported non-cavitary nodules or consolidation. And the No 6 (4) study reports the first case of disseminated *Emergomyces orientalis* infection in a Tibetan kidney transplant recipient, demonstrating that mNGS enables early and accurate diagnosis, fluconazole is ineffective, and posaconazole combined with flucytosine plus temporary immunosuppression withdrawal achieves clinical stability. mNGS can simultaneously detect almost all types of pathogens in the sample, including bacteria, fungi, viruses, parasites, etc., covering over 20,000 types of pathogenic microorganisms. There is no need to preset the detection target. mNGS also has some drawbacks: high detection costs, complex fee items, uneven supply of detection resources, limited clinical accessibility, prominent contamination of specimens and environmental nucleic acids, high risk of false positive/false negative reports, complex data analysis, highly dependent on professional experts in pathogenic microorganisms for interpretation, and can only clearly identify the species of pathogenic bacteria, but cannot directly provide results on the sensitivity of antifungal drugs (5). The price is low and it is particularly good at identifying difficult-to-culture, obligate anaerobic bacteria, rare pathogens, breaking through the bottleneck of traditional culture methods where “less than 1% of microorganisms can be cultured”. It can also detect mixed infections, avoiding missed diagnoses caused by single detection. The case4 (6) report identifies that *Emergomyces orientalis* can cause pulmonary infection in immunocompetent individuals with coal dust exposure, is accurately diagnosed by mNGS of bronchoalveolar lavage fluid, and can be effectively controlled with amphotericin B cholesterol sulfate complex followed by voriconazole despite transient renal impairment. The reports the world’s first case of concurrent mediastinal infection with *Emergomyces orientalis* and Mycobacterium fortuitum in an immunocompetent young woman, demonstrating that EBUS-guided biopsy combined with mNGS enables accurate identification of mixed rare pathogens, and tailored sequential antifungal-antibacterial combination therapy can achieve effective disease control (7). The 6 reported cases of infection have been compared (Table 1). A review systematically summarizes the taxonomy, epidemiology, clinical manifestations, diagnosis, and treatment of *emergomycosis* caused by species, highlights the key role of molecular sequencing in diagnosis, recommends amphotericin B plus itraconazole as the main therapeutic regimen, and prospects novel antifungal agents for future application (8).

**TABLE 1:** Comparison of 6 reported cases of infection with *Emergomyces orientalis.*

No.	Age/Gender	Immune status	Main clinical manifestations	Treatment regimen	Outcome
1 (1)	64/M	Type 2 diabetes	Fever, cough, expectoration; generalized pustular skin lesions, subcutaneous nodules; multiple nodules in the left lung	Amphotericin B induction and fluconazole → itraconazole sequential therapy, total 8 months	Cured, no recurrence at 10-year follow-up
2 (5)	51/M	Type 2 diabetes	Back pain, pharyngeal discomfort, scant sputum; multiple pulmonary nodules with cavitation	Oral itraconazole	Symptoms improved, cavitary lesions reduced; lost to follow-up
3 (7)	21/F	Immunocompetent (outdoor soil exposure)	Cough, fever; mediastinal mass/lymphadenopathy	Initial amphotericin B alone (effective); recurrence with *Mycobacterium fortuitum* coinfection → combined antifungal and anti-NTM therapy	Lesions markedly shrunk, disease controlled
4 (9)	52/M	Systemic lupus erythematosus with long-term steroids/immunosuppressants (severe immunocompromise)	Cough, expectoration, fever, headache; small pulmonary nodule in the right lung, mediastinal lymphadenopathy	Amphotericin B induction → oral itraconazole	Symptoms significantly improved, stable condition
5 (6)	52/M	Immunocompetent (coal mine dust exposure)	Intermittent fever, night sweats, cough, expectoration; multiple soft-tissue nodules in both lungs	Amphotericin B colloidal dispersion → itraconazole (poor response) → voriconazole	Lesions significantly absorbed, symptoms controlled
6 (4)	41/M	Kidney transplant recipient + immunosuppressants	Progressive right lower chest pain, mild cough with sputum; pulmonary consolidation, later abscess in the waist	Itraconazole → posaconazole with flucytosine,immunosuppressants tapered	Clinically stable, lesions improved

Due to the limited application of microbial metagenomic detection, some cases of *Emergomyces orientalis* have been missed. This fungus mainly exists in wild animals such as rodents, and infections occur more frequently in individuals with compromised immune function, such as HIV-infected individuals, organ transplant recipients, and those who have been using immunosuppressive agents for a long time (10). Previously, *Emergomyces* species were mainly associated with asymptomatic colonization of rodent lungs and were only rarely noted in human adiaspiromycosis, a lung infection characterized by large resting spores in tissues (11). However, this patient w*as* HIV infected who typically presented with high fever, chest pain, and multiple system involvement. Although traditional histological staining can detect fungal spores and is prone to confusion with histoplasma, cryptococcus, etc., (1). Some previous cases were missed due to the lack of genetic testing, misdiagnosis, and delayed treatment.

Currently, there is no unified treatment guideline for *Emergomyces orientalis* infection, and treatment is mostly based on observational studies and expert experience. Commonly used drugs include azole antifungal drugs and amphotericin B drugs. There have been reports that patients infected with *Emergomyces orientalis* did not respond to intravenous fluconazole and oral administration of levofloxacin (6). Immunocompromised patients often need to be treated with combination therapy, with a long course (up to several months), while immunocompetent patients can shorten the course appropriately based on the condition. After using amphotericin B lipid complex for anti-infection treatment, the patient’s pulmonary symptoms improved form our research and have not used azole.

## 4 Conclusion

In conclusion, pulmonary infection by *Emergomyces orientalis* is rare. Routine pathogenological examinations are prone to missed diagnoses. When encountering pneumonia patients with poor anti-infection treatment outcomes, especially when suspected of rare pathogen infection, mNGS detection should be promptly used to clarify the diagnosis. Treatment should be individualized based on the patient’s immune status to improve the prognosis. At the same time, more awareness of this disease is needed, and more clinical cases should be accumulated to provide a basis for the formulation of a unified diagnosis and treatment guideline.

## Data Availability

The metagenomic sequencing data (mNGS) supporting this study has been deposited in Zenodo and is publicly available at https://zenodo.org/records/21766186. All host data have been de-identified.
